# Lessons learned from youth-engaged research methods to conduct qualitative analysis with a transgender and gender-diverse youth advisory board

**DOI:** 10.1371/journal.pone.0355110

**Published:** 2026-07-30

**Authors:** An H. Pham, Zoe Webster, Melanie K. Bean, Suzanne Mazzeo, Kym Ahrens, Maria Thomson

**Affiliations:** 1 Department of Pediatrics, Virginia Commonwealth University, Richmond, Virginia, United States of America; 2 Department of Psychology, Virginia Commonwealth University, Richmond, Virginia, United States of America; 3 Department of Pediatrics, University of Washington, Seattle, Washington, United States of America; 4 Department of Health Behavior and Policy, Virginia Commonwealth University, Richmond, Virginia, United States of America; Nagoya University: Nagoya Daigaku, JAPAN

## Abstract

Although community-engaged research is becoming more common, youth-engaged research methods, particularly its application to qualitative analysis, remains rare. The Research on Identity Specific Eating Disorder Symptoms (RISES) Project developed, partnered with, and maintained a youth advisory board of five transgender, non-binary, and gender-diverse youth in all stages of a qualitative study, including qualitative analysis. Youth advisory board members contributed to a codebook, coded two to six transcripts each, provided feedback on themes and supporting quotes, and authored this paper. After one year, we achieved a retention rate of 83.3% of all youth advisory board members, and 100% of youth advisory board members trained in qualitative analysis. This paper describes the lessons learned from partnering with and training a transgender, non-binary, and gender-diverse youth advisory board to conduct qualitative analysis.

## Introduction

Traditionally, youth have been included in research as participants, and not collaborators. Youth-engaged research (YER) is a type of community-engaged research that integrates the lived realities of young people [1]. In community-engaged research broadly, research teams collaborate with community members, aiming to account for the power structures within group dynamics, maintain respect for the lived experiences of group members, and gain perspectives relevant to the research topic [2–4]. Engaging youth as collaborators via YER also builds capacity, taking action to empower their perspectives and provide youth with skills, confidence, and social engagement. The youth, in turn, provide the investigators with critical information regarding the feasibility and ecological validity of their work [1]. Additionally, the value of collaboration is generative. YER facilitates ways of thinking and lines of inquiry that would not be possible without their input. YER is especially critical to guide recruitment of understudied, minoritized youth populations and improve their representation in research to address gaps in science and health inequities.

Over the past 30 years, research with transgender, nonbinary, and gender diverse (TGD) youth has increased substantially; however, very little of this work implements YER methods [5–7]. YER, in general, is particularly useful for youth populations that have had a history of institutional abuse, such as TGD youth [8–13]. YER is necessary to create accurately informed care for TGD youth, yet pervasive discrimination against TGD individuals results in mistrust in medical and academic institutions; this must be accounted for in research settings [1]. A systematic review of participatory TGD youth research studies conducted between 2006 and 2022 identified themes of TGD youth empowerment and the desire to address systematic inequities across studies. Also, youth advisory boards (YAB) had a significant impact on research projects, including assistance in study design and participant recruitment [13]. Community-engaged researchers suggest that community members be honored as equal partners in all stages of research [14,15]. However, to our knowledge, there are no studies that partner with youth to conduct qualitative analyses. This study addresses this research gap and describes lessons learned from partnering with and training a YAB of TGD youth to conduct qualitative analysis.

## Materials and methods

### Research on identity specific eating disorder symptoms (RISES) project

RISES aims to advance our understanding of disordered eating among TGD youth to improve the prevention and treatment of disordered eating behaviors (i.e., restrictive eating, binge eating, laxative abuse, compulsive exercise) and eating disorders among TGD youth. To improve recruitment protocols of our studies with TGD youth, we conducted a qualitative study exploring barriers and promoters of research participation among 21 TGD youth aged 12–22 years old in 2025. In all stages of this study, from recruitment to data dissemination, we partnered with a YAB of five TGD youth. This report focuses on the YER methods implemented to conduct qualitative analysis with this YAB.

### Research team

The primary investigator (PI) is a cisgender woman who is a gender-affirming Adolescent Medicine physician with more than 8 years of experience conducting research with TGD youth. As described by Katz-Wise et al. in their article describing the lessons they learned from their community-based participatory research (CBPR) study with TGD youth, a challenge commonly faced in community-engaged research is insider/outsider status between the researchers and the community affected by the research [10]. To address this challenge, we prioritized representation of TGD people on our research team. The YAB consisted of five TGD youth (see below for additional details). Qualitative interviews were conducted by two TGD research staff members, the research coordinator and community consultant. The community consultant is a mental health provider and has expertise in disordered eating among TGD people. YAB members were mentored by seven graduate/medical students, four of whom identify as TGD. Two additional TGD research members, both medical students, assisted with coding and thematic analysis.

### Youth advisory board (YAB)

#### Recruitment.

The recruitment and maintenance of our study’s YAB was guided by the three key principles of Youth Participatory Action Research (YPAR): 1) this study is informed by the lived experiences TGD youth, 2) YAB members are collaborators on all aspects of this qualitative study, and 3) the purpose of our qualitative study on TGD youth research participation is to advance research that improves the lives of TGD youth [16]. We created an initial qualitative study protocol based on our collective experience conducting qualitative analysis and working with community and youth advisory boards. From 08/04/2024, we recruited TGD youth aged 16–21 years with a history of disordered eating to join a YAB and act as advisors across all RISES research activities. Because the RISES project is a multi-study project, recruitment of our YAB is ongoing. A history of disordered eating was satisfied if a potential YAB member selected “yes” to questions 1, 2, 7, 8, 9, or 10 on the Eating Disorder Examination Questionnaire-Short (EDE-QS; measure of eating pathology that has been validated in a sample of 71 TGD young adults) [17]. The selected items measure disordered eating behaviors, not cognitions, because cognitions related to body and shape dissatisfaction may be secondary to gender dysphoria and not eating pathology. Virginia Commonwealth University’s Institutional Review Board approved our research protocol prior to recruitment of YAB members (HM20026881).

We asked TGD youth organizations and gender-affirming medical and mental health providers to 1) post physical recruitment flyers at their organizations and/or 2) email an electronic recruitment flyer directly to the TGD youth and families of TGD youth they serve. The recruitment flyer included a QR code to a REDCap survey with the EDE-QS and demographic questions including age, gender identity, US state of residence, race/ethnicity [18,19]. After completing the screening survey, we invited the potential YAB member to a Zoom video interview with the TGD research coordinator. In the interview, we asked potential YAB members about their experiences with community advocacy and how they would like to contribute to community advocacy. We also asked potential YAB members to reflect on participating in a longitudinal research project on eating pathology given their personal history of disordered eating behaviors. After the interview, the research coordinator emailed potential YAB members with a follow-up task. We asked potential YAB members to review the National Eating Disorders Association (NEDA) and Trevor Project websites on eating disorders in sexual and gender minority populations, and then answer reflective questions on the websites’ information (i.e., What did you find most surprising? What kinds of questions do you have about how the data was collected, analyzed, or shared?). The follow-up task had two purposes. It provided an opportunity to decrease YAB attrition. Additionally, the inclusion of both a formal interview and a follow-up email task in the recruitment process provided two separate opportunities for potential YAB members to showcase their strengths and motivations to participate in a YAB. This was crucial, as many youth have never participated in a professional interview, and many TGD youth experience social anxiety [20].

Due to the time commitment and ongoing discussions of disordered eating as a YAB member, we required parental consent for all YAB members under 18 years of age. Twelve people completed the screening survey, 8 completed the interview and follow-up task, and we invited 6 TGD youth to provide written consent/assent and join our YAB. We did not invite two TGD youth to join our YAB because they were experiencing high levels of distress from their disordered eating and there were concerns with stress from participating in ongoing discussions on disordered eating research. One YAB member discontinued their participation after one month due to difficulties managing YAB responsibilities as a college student with a job. Throughout our qualitative research study on research participation among TGD youth, we worked closely with five TGD YAB members. At the time of recruitment, 3 members were 18 years of age and 2 were 16 years of age. Two identified as transmasculine, one as transmasculine/non-binary, one as transfeminine, and one as non-binary. Four YAB members described themselves as white and three as Hispanic. Four YAB members were in Virginia and one in California.

#### Responsibilities.

The five YAB members provided input on every aspect of our qualitative study. Table 1 lists the YAB responsibilities for each research phase. YAB members were free to choose the responsibilities that they participated in. Virtual Zoom YAB meetings occurred 1–2 times a month. These meetings included training sessions as described below. At the first YAB meeting, we established community agreement and guidelines. This included shared expectations for maintaining an inclusive, affirming, and safe YAB environment, and guidelines for participation (i.e., if a YAB member missed 3 meetings we would meet with the YAB member to discuss whether they should continue on the YAB). All responsibilities were conducted virtually or asynchronously. To evaluate participation and engagement in this study, we measured the number of YAB meetings attended, involvement in qualitative analysis (whether YAB member contributed to the codebook and how many transcripts they coded), and partnership building with the brief version of the Research Engagement Survey Tool (REST) [21]. YAB members were compensated $125 for every 3 months of participation. Additionally, we compensated YAB members $10 for each transcript they coded during qualitative analysis.

**Table 1:** Responsibilities of YAB Members.

Recruitment
Provide feedback on recruitment flyers
Provide recommendations on where to recruit TGD youth
Recruit known TGD youth contacts
Data collection
Contribute questions to the qualitative interview guide
Provide feedback on drafts of qualitative interview guide
Pilot qualitative interview guide
Qualitative data analysis
Contribute to codebook
Code transcripts
Review and finalize themes
Provide feedback on exemplary quotes for manuscript
Data dissemination
Contribute to manuscript as an author
Contribute to videos and infographics for data dissemination to the community (i.e., RISES social media, TGD and eating pathology organizations)

### Training

We provided training in the responsible conduct of research prior to the start of our qualitative study recruitment. Every YAB member completed Society of Behavioral Medicine’s “NIH Good Clinical Practice Training For Social and Behavioral Research” course. The PI led three 45-minute virtual didactic training sessions on general research, qualitative research, and qualitative analysis. Following completion of these formal didactic sessions, YAB members applied this knowledge to hands-on coding practice. The research coordinator asked the YAB members their favorite hobbies. The 2 most popular responses were anime and video games. The PI made up a qualitative study on coping during the COVID-19 pandemic shutdown among adolescents and wrote four interview transcripts that weaved anime and video games into the responses. During the YAB meeting, YAB members practiced qualitative analysis by: 1) reading 2 transcripts to become familiar with the data, 2) using those same 2 transcripts to create a codebook, and 3) coding the remaining 2 transcripts using the codebook and modifying the codebook as needed throughout this coding process. In addition to formal didactic and hands-on training, each YAB member was paired with at least one mentor, a graduate/medical student trained in qualitative analysis. As described below, YAB mentors provided ongoing mentoring on qualitative analysis throughout the coding process.

For the YAB mentors, the PI briefed all mentors at a virtual meeting to describe expectations and responsibilities. Throughout the qualitative study, the PI and research coordinator provided ongoing group and individual YAB mentor support (see below for specific examples). Lastly, the PI provided relevant materials and resources for specific aspects of mentorship (i.e., mentoring research writers) [22].

### Qualitative Analysis Process

Prior to conducting qualitative analysis, we provided the YAB with individual mentor descriptions, including their gender identity, research experience, and hobbies. We gave YAB members the option of choosing their mentor based on these descriptions. After pairing YAB members and mentors, they met by phone or Zoom to introduce themselves, identify best methods to contact each other, find best times of the week to check in with each other, discuss working and communication styles, and anything else that would be helpful for their mentorship relationship. For our qualitative research study on research participation among TGD youth, we applied Braun and Clark’s 6 steps of reflexive thematic analysis [23,24]. For Step 1, YAB members/mentors read the same 2 transcripts to familiarize themselves with the data. To generate initial codes in Step 2, YAB members/mentors developed a codebook by independently coding the same 5 transcripts. The PI then compiled these codes to create a codebook. The PI adjusted code definitions, clarified code definitions, and added/combined codes as needed throughout the coding process. Four of the 5 YAB members contributed to the codebook because one YAB member required additional time and support to complete the “NIH Good Clinical Practice Training for Social and Behavioral Research” online course. Once the codebook was developed, each transcript was coded by 2 research team members, including YAB members with the assistance of their mentor(s). The initial plan was for all transcripts to be double coded by YAB members/mentors; however, given the time needed for YAB members to code each transcript, we assigned the remaining transcripts to TGD research team members with experience in coding. This shift allowed all transcripts to be coded by at least 2 coders who are TGD, while accomplishing our goal of completing coding within a 3-month period. The YAB members/mentors coded 25 out of 42 transcripts (21 transcripts that were double coded for a total of 42 transcripts). The PI compared codes for each transcript and resolved discrepancies by discussion with YAB members/mentors. Using a data driven, inductive approach, the PI and two TGD research team members reviewed patterns and established preliminary themes for Step 3. Originally, we planned for the YAB members to review patterns and establish themes. However, YAB mentors and the research coordinator recommended the YAB members take a break from qualitative analysis due to the amount of learning and effort they contributed to coding. Instead, the research coordinator presented the preliminary themes to the YAB, and they provided feedback and confirmed consistent themes (Steps 4–5). To write the report in Step 6, the YAB members provided feedback on exemplary quotes to include in the manuscript and were invited to author the manuscript on the qualitative findings. One of the YAB members contributed to the manuscript and met authorship criteria per International Committee of Medical Journal Editors (ICMJE) authorship criteria with the assistance of their YAB mentor.

### Lessons learned

We have gained knowledge throughout this process of qualitative analysis using YER methods with a YAB of five TGD youth. Based on this experience, we have compiled the following communication, responsibilities, and engagement recommendations from feedback from the research team, specifically YAB members, YAB mentors, and our research coordinator.

### Communication

Communication is the most difficult challenge when working longitudinally with a TGD YAB. All of our YAB members are students and/or employed, and their schedules vary week to week. The main form of communication between the research coordinator and YAB members was group messaging via Signal. For safety/privacy reasons, we switched from GroupMe to Signal because of Signal’s strong end-to-end encryption. Group messaging increased YAB members’ participation and promoted group camaraderie and team building. YAB members found a group messaging phone application more convenient than group e-mail. Initially, YAB members frequently forgot about YAB meetings. In response, the research coordinator sent reminder messages to the Signal group chat the morning of and an hour before YAB meeting times, which resulted in better attendance.

Originally, we planned for YAB members to meet by Zoom bi-weekly during the coding process to discuss any coding questions or concerns. However, YAB members were overwhelmed with communication with their mentors while coding. Thus, to minimize burden on YAB members, the research coordinator instead sent a weekly message to the Signal group chat to give YAB members the opportunity to provide coding tips and/or ask questions to the group. Since the YAB members’ experiential training relied primarily on their communication and learning with their mentor(s), this strategy allowed mentors to build stronger relationships with their YAB mentees. Our research coordinator acted as a liaison and facilitator for the YAB members and their mentors. Some mentors reported difficulties contacting their YAB mentee. To address these challenges, the research coordinator, who had worked with the YAB for 6 + months, offered mentors tips for communicating with their mentees. These tips were personalized to their paired YAB mentee.

During the year we conducted our qualitative study, the US President signed multiple executive orders that directly impacted TGD youth. [25] We were mindful of how these executive orders affected our YAB members, and maintained a strong connection with them throughout this time. We checked in with them to see if they wanted to pause their responsibilities or continue as planned. All YAB members wanted to continue with research activities because: 1) they wanted a distraction during these stressful times, and 2) they found comfort in spending time with other TGD youth. We also asked YAB members if we could be of any support or assistance during these difficult times. In response, TGD YAB members remarked “*Thank you so much, I am so grateful to have you guys as support!!*”, “*Thank you so much, that means a lot to me*,” and “*I really, really appreciate i*t.”

### Responsibilities

We provided asynchronous opportunities to complete non-coding tasks (i.e., providing feedback on an interview script and other research materials). However, in general, YAB members preferred completing their work in YAB meetings, and expressed the desire to minimize asynchronous work. With respect to coding, YAB members either coded with their YAB mentor(s) together at the same time or coded the same transcripts asynchronously and then provided explanations for their codes. For the YAB member/mentor(s) who coded transcripts independently, many pairs used Google shared documents to facilitate asynchronous work. To enhance the rigor of the coding process as well as build capacity for YAB members, we emphasized the importance of mentorship relationships to the research team. YAB mentors were expected to check in with their YAB mentee at least once a week. Every week, the PI emailed YAB mentors, and the research coordinator sent a group chat to YAB members to discuss questions regarding codes, coding, and mentorship relationships. Each YAB member and their mentor(s) coded a different number of transcripts. This allowed YAB members to balance coding and individual time commitments at the time of coding, such as exams and other personal circumstances (i.e., moving). Each YAB member coded 2–6 transcripts. Additionally, the research coordinator offered individual assistance to YAB members. For example, one YAB member struggled to complete their “NIH Good Clinical Practice Training for Social and Behavioral Health” course due to technical issues and school exams, which delayed their ability to start coding transcripts. The research coordinator set up a Zoom meeting with this YAB member to resolve the online course issue, and the YAB member began coding transcripts after the completion of the school year (6 weeks after the other YAB members started coding). This additional support and flexibility gave the YAB member the opportunity to gain coding skills during this research study.

### Engagement

We achieved a YAB retention rate of 83.3% after one year. The one YAB member who withdrew their participation did so 1 month after signing consent, and did not participate in qualitative analysis. This member left because of their overwhelming work and school schedule, and they indicated interest in participating as a research participant in future work. We attribute our high YAB retention rate to our investment in incorporating YAB input into research protocols. For example, we originally provided $250 for every 6 months of participation. After feedback from the research coordinator and YAB members that YAB members would benefit from more frequent compensation, we changed the payment schedule to $125 for every 3 months. Throughout the 3-month coding process, we kept detailed notes on recommendations and critiques from YAB members and mentors. We used that feedback to make changes to our qualitative analysis processes. We additionally asked YAB mentors to provide individual feedback on the strengths and areas of improvement for their YAB mentee. We compiled this information and conducted individual meetings with each YAB member to provide a summary evaluation of their participation in this research study. We also used this meeting time to gather feedback from YAB members. We will incorporate this feedback into future research protocols with our YAB.

Another approach to maintaining engagement was harnessing the YAB’s motivation to participate. Every few months, in addition to reminding the YAB of expectations for participation (i.e., attending meetings, responding to the research coordinator in a timely manner), we asked YAB members to remind the group of their motivation for joining the RISES team. Of note, when re-iterating expectations, we provided more detailed descriptions by email and at YAB meetings, and then concise summaries via group chat messaging. In addition, every time the YAB contributed to a research material (i.e., providing feedback on the recruitment flyer, creating questions for the interview guide), we showed the YAB the final research material to report back, bolster their sense of pride and achievement, and provide recognition of their input.

To create an affirming research environment that prioritizes open communication and transparency, we continuously considered power imbalances within our research team. Firstly, YAB members were mostly in contact with a research coordinator who is a TGD young adult with experience working professionally with youth. The PI only interacted with YAB members during research training sessions, and prior to these sessions she was forthright about pertinent aspects of her identity, including being cisgender, thin, a gender-affirming medical provider, and an academician at a large university. When assigning YAB members with their mentors, we also gave YAB members the option to choose their mentor based off their gender identity and specific research interests. Additionally, YAB members were given the opportunity to provide feedback on the research team and research processes through one-on-one meetings with the research coordinator and REDCap surveys [18,19]. Lastly, it was important for all research team members to acknowledge the unique power that our YAB members hold with their own skills and experience as youth directly affected by the research we conduct.

Lastly, in addition to the rigor of our research, extensive training prior to the start of qualitative analysis was crucial for not only engagement, but also capacity building, one of our main priorities for youth-engagement. Although youth engagement allows research teams to learn from community youth members to improve research protocols, bidirectional learning promotes equity, sustained YAB partnerships, and YAB knowledge and skills. In addition to research training, we have contributed to YAB professional development in multiple ways. We encouraged YAB mentors to provide mentorship outside of coding. For example, one YAB mentor provided advice on college applications and submitted a college application letter of recommendation for their mentee. We have also dedicated specific YAB meetings to professional development topics, including building curriculum vitae/resumes and preparing for job interviews. Our hope is that this YAB ultimately contributes to better TGD representation among professionals who conduct research. In the shorter-term, we have empowered YAB members to apply knowledge and utilize new skills outside of our research project, in both academic and non-academic settings.

## Conclusions

YER continues to be rare within health research, particularly engagement of youth community members in the analysis process of research studies. We trained five YAB members to conduct qualitative analysis as coders with a retention rate of 100% of the trained coders. Each YAB member coded 2–6 transcripts, all members provided feedback on themes and supporting quotes, and one YAB member is an author on this paper. This is evidence that with resources dedicated to communication, engagement, and clear responsibilities, YER methods can be applied successfully to qualitative analysis.

Our results indicate that monetary funding and time are specific resources that enhanced engagement. YABs should be compensated for their time and effort. To offset financial burden, graduate and medical students dedicated to improving the lives of TGD youth through research volunteered to mentor YAB members. Many of our YAB mentors volunteered their time in areas outside of this specific study, including professional development.

We also found that flexibility, clear expectations, and genuine investment in our YAB’s wellness were crucial to the success of our timeline. To stay within our general timeline while also preventing the YAB members from feeling overwhelmed during qualitative analysis, we continuously gathered feedback from the YAB members/mentors and adjusted their responsibilities within our research protocol. We also frequently reminded the YAB of expectations, provided information on the research process so that the YAB understood how the completion of their tasks contributed to the overall timeline, and asked YAB members to remind themselves and the group of their motivations for participating on the RISES research team. We found that if the YAB members were continuously learning, discovering how their newfound skills were contributing to the success of our research, and enjoying their time, they not only continued their participation, but the efficiency and quality of their work were also positively affected.

Our partnership with a TGD YAB throughout qualitative analysis strengthened the rigor and validity of our results, and cultivated research skills and professional development of five TGD youth. We were also given the unique opportunity to create a research environment where academicians supported five TGD youth who also provided support for each other during a politically difficult year for TGD communities. We acknowledge that youth-engaged research methods are resource intensive and require flexibility, but there are 1) clear benefits to this commitment and 2) varying levels of youth engagement that can be applied to research. We hope that the lessons we have learned in our qualitative study can be adapted to the unique resources and needs of different types of research studies involving youth. We will continue to learn from our YAB and apply the lessons learned to future research protocols with our current and expanding YAB.
