# Supplementary material for: Lessons learned from youth-engaged research methods to conduct qualitative analysis with a transgender and gender-diverse youth advisory board
Source: PLoS One. 2026 Jul 30;21(7):e0355110. doi: 10.1371/journal.pone.0355110 (PMC13422823; doi:10.1371/journal.pone.0355110)
Supplement: Attachment — Submitted filename: Response to Reviewers.7.10.docx [file pone.0355110.s002.docx]

Thank you for taking the time to provide thoughtful reviews of our manuscript, “Lessons learned form youth-engaged research methods to conduct qualitative analysis with a transgender and gender-diverse youth advisory board”. We have made edits based on the feedback provided and believe they have strengthened this manuscript.  Here is our response to each item of feedback organized by reviewer:

Reviewer #1:

- The only unclear part of the manuscript was in line 138 "there were concerns with a long-term commitment to disordered eating research." - was this a requirement for YAB recruitment? I didn't see it in the above recruitment part.
  - Response: Thank you for the opportunity to clarify this sentence. We have edited this sentence because a long-term commitment was not a requirement. The sentence now reads, “We did not invite two TGD youth to join our YAB because they were experiencing high levels of distress from their disordered eating and there were concerns with stress from participating in ongoing discussions on disordered eating research.”
    - See lines 144-147 of the clean manuscript

Reviewer #2:

- A stronger methodological framework would be helpful. Currently the manuscript describes activities but does not clearly theorize the engagement approach. (e.g., Hart's Ladder of Participation, Youth Participatory Action Research, CBPR principles, or another engagement framework)
  - Response: We appreciate the reviewer’s thoughtful recommendation, and the addition of this information has strengthened our manuscript. Our research process was guided by Youth Participatory Action Research. We have added this sentence to the methods section, “The recruitment and maintenance of our study’s YAB was guided by the three key principles of Youth Participatory Action Research (YPAR): 1) this study is informed by the lived experiences TGD youth, 2) YAB members are collaborators on all aspects of this qualitative study, and 3) the purpose of our qualitative study on TGD youth research participation is to advance research that improves the lives of TGD youth.(16)”
    - See lines 104-108 of the clean manuscript.
- A section or thoughts on reflexivity. While information is added on the PI, there is little beyond the facts and not much to discuss how power dynamics were approached, how differences in age, professional status, race, socioeconomic status, and education may have influenced interactions; how the team mitigated tokenism and power imbalances
  - Response: We agree with the reviewer that this is very important information. We have added a paragraph within the Engagement subsection to describe the steps we took to address power imbalances between the YAB and other research team members.
    - See lines 318-330 of the clean manuscript
- What changed because youth were involved,
  - Response: Throughout the manuscript we have noted what is unique to youth advisory board members. For example, for YAB members under 18 years of age, we required legal guardian consent. We gave potential YAB members two separate opportunities to showcase their interest in skills, an interview and a follow-up task because many youth have not participated in formal interviews. We also required flexibility due to many youth members juggling responsibilities of being students. This included additional one-on-one support for one youth member and a delay in coding due to their studying for finals. Another example is applying youth chosen topics (anime and video games) into practice coding sessions.
- How youth themselves evaluated the experience.
  - Response: YAB members provided their feedback through one-on-one feedback sessions with the research coordinator and a REDCap survey that included the Research Engagement Survey Tool (REST) and open-ended questions about their participation and our research processes.
    - See lines 306-308, 326-328 of the clean manuscript
- Abstract: Partnered with - was the advisory group already in existence and if not how was it formed and recruited? (I see you talk about the recruitment and creation of the advisory group in the methods section, but would be helpful to have a slight nod to it or brief information in abstract since creation of a YAB is different than using a previously created YAB)
  - Response: We have edited the sentence to clarify that we developed this YAB as opposed to partnering with an already established YAB. The sentence now reads, “The Research on Identity Specific Eating Disorder Symptoms (RISES) Project developed, partnered with, and maintained a youth advisory board of five transgender, non-binary, and gender-diverse youth in all stages of a qualitative study, including qualitative analysis.”
    - See lines 38-41 of the clean manuscript
- Introduction: line 50 - add citation
  - Response: We have added a citation.
    - See the citation for the second sentence of the first paragraph of the introduction.
- Methods: YAB demographics would be nice
  - Response: We have added the YAB’s age, race/ethnicity, gender identity, and state.
    - See lines 150-154 of the clean manuscript
- Methods: Were trainings in addition to the 1-2 YAB meetings per month or some months the meetings were trainings?
  - Response: These trainings were included in the 1-2 YAB meetings per month. We added a sentence to clarify this.
    - See line 159 of the clean manuscript
- Methods: Qualitative analysis process line 206 - was the discussion with the YAB or research team?
  - Response: These discrepancy discussions were with the YAB members/mentors. We have added this to qualitative analysis section.
    - See lines 217-218 of the clean manuscript
- For all of your lessons learned - how would you recommend someone else utilize these lessons?
  - Response: We were intentional on not listing specific recommendations since we acknowledge that high levels of youth engagement in research rely on resources, specifically time and money. Because of this, we provided a variety of examples describing how we maintained high levels of engagement throughout our qualitative analysis process so that readers can consider applying varying levels of youth engagement to their research. We have modified our closing paragraph to reflect this. It now reads, “We acknowledge that youth-engaged research methods are resource intensive and require flexibility, but there are 1) clear benefits to this commitment and 2) varying levels of youth engagement that can be applied to research. We hope that the lessons we have learned in our qualitative study can be adapted to the unique resources and needs of different types of research studies involving youth. We will continue to learn from our YAB and apply the lessons learned to future research protocols with our current and expanding YAB.”
    - See lines 374-380 of the clean manuscript
- Communication: line 251 - I wonder if you can speak more to the joint benefit of YAB - while they were informing the research they were also building community amongst themselves with support from allies in positions of power
  - Response: Thank you for this special point. We have added a sentence to our closing paragraph about this. Our closing paragraph now reads, “Our partnership with a TGD YAB throughout qualitative analysis strengthened the rigor and validity of our results, and cultivated research skills and professional development of five TGD youth. We were also given the unique opportunity to create a research environment where academicians supported five TGD youth who also provided support for each other during a politically difficult year for TGD communities.”
    - See lines 370-374 of the clean manuscript
