# Supplementary material for: Lessons learned from youth-engaged research methods to conduct qualitative analysis with a transgender and gender-diverse youth advisory board
Source: PLoS One. 2026 Jul 30;21(7):e0355110. doi: 10.1371/journal.pone.0355110 (PMC13422823; doi:10.1371/journal.pone.0355110)
Supplement: Peer review history

# Peer review: Decision Letter 0

**DOI:** 10.1371/journal.pone.0355110.r001

**Authors:** Branko Aleksic

9 Jul 2026

PONE-D-26-08402

Lessons Learned From Youth-Engaged Research Methods to Conduct Qualitative Analysis with a Transgender and Gender-Diverse Youth Advisory Board

PLOS One

Dear Dr. Pham,

Thank you for submitting your manuscript to PLOS ONE. After careful consideration, we feel that it has merit but does not fully meet PLOS ONE’s publication criteria as it currently stands. Therefore, we invite you to submit a revised version of the manuscript that addresses the points raised during the review process.

**Very nice paper.  See additional comments below.**

Please submit your revised manuscript by Aug 23 2026 11:59PM. If you will need more time than this to complete your revisions, please reply to this message or contact the journal office at plosone@plos.org. When you're ready to submit your revision, log on to https://www.editorialmanager.com/pone/ and select the 'Submissions Needing Revision' folder to locate your manuscript file.

Please include the following items when submitting your revised manuscript:

- A letter that responds to each point raised by the academic editor and reviewer(s). You should upload this letter as a separate file labeled 'Response to Reviewers'.
- A marked-up copy of your manuscript that highlights changes made to the original version. You should upload this as a separate file labeled 'Revised Manuscript with Track Changes'.
- An unmarked version of your revised paper without tracked changes. You should upload this as a separate file labeled 'Manuscript'.

If you would like to make changes to your financial disclosure, please include your updated statement in your cover letter. Guidelines for resubmitting your figure files are available below the reviewer comments at the end of this letter.

If applicable, we recommend that you deposit your laboratory protocols in protocols.io to enhance the reproducibility of your results. Protocols.io assigns your protocol its own identifier (DOI) so that it can be cited independently in the future. For instructions see: https://journals.plos.org/plosone/s/submission-guidelines#loc-laboratory-protocols. Additionally, PLOS ONE offers an option for publishing peer-reviewed Lab Protocol articles, which describe protocols hosted on protocols.io. Read more information on sharing protocols at https://plos.org/protocols?utm_medium=editorial-email&utm_source=authorletters&utm_campaign=protocols.

As the corresponding author, your ORCID iD is verified in the submission system and will appear in the published article. PLOS supports the use of ORCID, and we encourage all coauthors to register for an ORCID iD and use it as well. Please encourage your coauthors to verify their ORCID iD within the submission system before final acceptance, as unverified ORCID iDs will not appear in the published article. *Only*  the individual author can complete the verification step; PLOS staff *cannot*  verify ORCID iDs on behalf of authors.

We look forward to receiving your revised manuscript.

Kind regards,

Branko Aleksic, MD, PhD

Academic Editor

PLOS One

**Journal Requirements:**

1. When submitting your revision, we need you to address these additional requirements.

Please ensure that your manuscript meets PLOS ONE's style requirements, including those for file naming. The PLOS ONE style templates can be found at

https://journals.plos.org/plosone/s/file?id=wjVg/PLOSOne_formatting_sample_main_body.pdf and

https://journals.plos.org/plosone/s/file?id=ba62/PLOSOne_formatting_sample_title_authors_affiliations.pdf

2. We note that the grant information you provided in the ‘Funding Information’ and ‘Financial Disclosure’ sections do not match.

When you resubmit, please ensure that you provide the correct grant numbers for the awards you received for your study in the ‘Funding Information’ section.

3. Thank you for stating in your Funding Statement:

This work was supported by National Institutes of Health [K23MH134111 to A.P.]

Please provide an amended statement that declares *all* the funding or sources of support (whether external or internal to your organization) received during this study, as detailed online in our guide for authors at http://journals.plos.org/plosone/s/submit-now.  Please also include the statement “There was no additional external funding received for this study.” in your updated Funding Statement.

Please include your amended Funding Statement within your cover letter. We will change the online submission form on your behalf.

4. Thank you for stating the following financial disclosure:

This work was supported by National Institutes of Health [K23MH134111 to A.P.]

Please state what role the funders took in the study.  If the funders had no role, please state: "The funders had no role in study design, data collection and analysis, decision to publish, or preparation of the manuscript."

If this statement is not correct you must amend it as needed.

Please include this amended Role of Funder statement in your cover letter; we will change the online submission form on your behalf.

5. We note that you have indicated that there are restrictions to data sharing for this study. For studies involving human research participant data or other sensitive data, we encourage authors to share de-identified or anonymized data. However, when data cannot be publicly shared for ethical reasons, we allow authors to make their data sets available upon request. For information on unacceptable data access restrictions, please see http://journals.plos.org/plosone/s/data-availability#loc-unacceptable-data-access-restrictions.

Before we proceed with your manuscript, please address the following prompts:

a) If there are ethical or legal restrictions on sharing a de-identified data set, please explain them in detail (e.g., data contain potentially identifying or sensitive patient information, data are owned by a third-party organization, etc.) and who has imposed them (e.g., a Research Ethics Committee or Institutional Review Board, etc.). Please also provide contact information for a data access committee, ethics committee, or other institutional body to which data requests may be sent.

b) If there are no restrictions, please upload the minimal anonymized data set necessary to replicate your study findings to a stable, public repository and provide us with the relevant URLs, DOIs, or accession numbers. Please see http://www.bmj.com/content/340/bmj.c181.long for guidelines on how to de-identify and prepare clinical data for publication. For a list of recommended repositories, please see https://journals.plos.org/plosone/s/recommended-repositories. You also have the option of uploading the data as Supporting Information files, but we would recommend depositing data directly to a data repository if possible.

Please update your Data Availability statement in the submission form accordingly.

6. Your ethics statement should only appear in the Methods section of your manuscript. If your ethics statement is written in any section besides the Methods, please move it to the Methods section and delete it from any other section. Please ensure that your ethics statement is included in your manuscript, as the ethics statement entered into the online submission form will not be published alongside your manuscript.

7. Please include your tables as part of your main manuscript and remove the individual files. Please note that supplementary tables (should remain/ be uploaded) as separate "supporting information" files.

8. If the reviewer comments include a recommendation to cite specific previously published works, please review and evaluate these publications to determine whether they are relevant and should be cited. There is no requirement to cite these works unless the editor has indicated otherwise.

9. Please review your reference list to ensure that it is complete and correct. If you have cited papers that have been retracted, please include the rationale for doing so in the manuscript text, or remove these references and replace them with relevant current references. Any changes to the reference list should be mentioned in the rebuttal letter that accompanies your revised manuscript. If you need to cite a retracted article, indicate the article’s retracted status in the References list and also include a citation and full reference for the retraction notice.

[Note: HTML markup is below. Please do not edit.]

Reviewers' comments:

Reviewer's Responses to Questions

**Comments to the Author**

1. Is the manuscript technically sound, and do the data support the conclusions?

The manuscript must describe a technically sound piece of scientific research with data that supports the conclusions. Experiments must have been conducted rigorously, with appropriate controls, replication, and sample sizes. The conclusions must be drawn appropriately based on the data presented.

Reviewer #1: Yes

Reviewer #2: Yes

**********

2. Has the statistical analysis been performed appropriately and rigorously?

Reviewer #1: Yes

Reviewer #2: N/A

**********

3. Have the authors made all data underlying the findings in their manuscript fully available?

The PLOS Data policy requires authors to make all data underlying the findings described in their manuscript fully available without restriction, with rare exception (please refer to the Data Availability Statement in the manuscript PDF file). The data should be provided as part of the manuscript or its supporting information, or deposited to a public repository. For example, in addition to summary statistics, the data points behind means, medians and variance measures should be available. If there are restrictions on publicly sharing data—e.g. participant privacy or use of data from a third party—those must be specified.

Reviewer #1: Yes

Reviewer #2: Yes

**********

4. Is the manuscript presented in an intelligible fashion and written in standard English?

PLOS ONE does not copyedit accepted manuscripts, so the language in submitted articles must be clear, correct, and unambiguous. Any typographical or grammatical errors should be corrected at revision, so please note any specific errors here.

Reviewer #1: Yes

Reviewer #2: Yes

**********

5. Review Comments to the Author

Please use the space provided to explain your answers to the questions above. You may also include additional comments for the author, including concerns about dual publication, research ethics, or publication ethics. (Please upload your review as an attachment if it exceeds 20,000 characters)

**Reviewer #1:**  Excellent paper on a much-needed topic. Paper is well-written and I personally learned new ways to engage youth in TGD research.

The only unclear part of the manuscript was in line 138 "there were concerns with a long-term commitment to disordered eating research." - was this a requirement for YAB recruitment? I didn't see it in the above recruitment part.

Otherwise, I appreciate and thank you for this work and getting this experience to the field. I especially liked the part where the team surveyed the YAB members on their interests (anime and video games) and then created mockup transcripts. This is an excellent way to engage youth who are also TGD and I will be using this method in my own work in the future.

**Reviewer #2:**  Overall this is a well written manuscript that adds a lot of interesting information and methodology to the field when working with YABs for qualitative research.

Major comments

A stronger methodological framework would be helpful. Currently the manuscript describes activities but does not clearly theorize the engagement approach. (e.g., Hart's Ladder of Participation, Youth Participatory Action Research, CBPR principles, or another engagement framework)

A section or thoughts on reflexivity. While information is added on the PI, there is little beyond the facts and not much to discuss how power dynamics were approached, how differences in age, professional status, race, socioeconomic status, and education may have influenced interactions; how the team mitigated tokenism and power imbalances

What changed because youth were involved,

How youth themselves evaluated the experience.

Minor edits

Abstract

Partnered with - was the advisory group already in existence and if not how was it formed and recruited?

(I see you talk about the recruitment and creation of the advisory group in the methods section, but would be helpful to have a slight nod to it or brief information in abstract since creation of a YAB is different than using a previously created YAB)

Introduction

line 50 - add citation

Methods

YAB demographics would be nice

Were trainings in addition to the 1-2 YAB meetings per month or some months the meetings were trainings?

Qualitative analysis process

line 206 - was the discussion with the YAB or research team?

For all of your lessons learned - how would you recommend someone else utilize these lessons?

Communication

line 251 - I wonder if you can speak more to the joint benefit of YAB - while they were informing the research they were also building community amongst themselves with support from allies in positions of power

**********

6. PLOS authors have the option to publish the peer review history of their article (what does this mean?). If published, this will include your full peer review and any attached files.

If you choose “no”, your identity will remain anonymous but your review may still be made public.

**Do you want your identity to be public for this peer review?**  For information about this choice, including consent withdrawal, please see our Privacy Policy.

Reviewer #1: **Yes:**  Darya Adams

Reviewer #2: **Yes:**  Amanda Cruce

**********

[NOTE: If reviewer comments were submitted as an attachment file, they will be attached to this email and accessible via the submission site. Please log into your account, locate the manuscript record, and check for the action link "View Attachments". If this link does not appear, there are no attachment files.]

To ensure your figures meet our technical requirements, please review our figure guidelines: https://journals.plos.org/plosone/s/figures

You may also use PLOS’s free figure tool, NAAS, to help you prepare publication quality figures: https://journals.plos.org/plosone/s/figures#loc-tools-for-figure-preparation.

NAAS will assess whether your figures meet our technical requirements by comparing each figure against our figure specifications.

# Author comment: Author response to Decision Letter 1

**DOI:** 10.1371/journal.pone.0355110.r002

13 Jul 2026

Thank you for taking the time to provide thoughtful reviews of our manuscript, “Lessons learned form youth-engaged research methods to conduct qualitative analysis with a transgender and gender-diverse youth advisory board”. We have made edits based on the feedback provided and believe they have strengthened this manuscript. Here is our response to each item of feedback organized by reviewer:

Reviewer #1:

- The only unclear part of the manuscript was in line 138 "there were concerns with a long-term commitment to disordered eating research." - was this a requirement for YAB recruitment? I didn't see it in the above recruitment part.

o Response: Thank you for the opportunity to clarify this sentence. We have edited this sentence because a long-term commitment was not a requirement. The sentence now reads, “We did not invite two TGD youth to join our YAB because they were experiencing high levels of distress from their disordered eating and there were concerns with stress from participating in ongoing discussions on disordered eating research.”

• See lines 144-147 of the clean manuscript

Reviewer #2:

- A stronger methodological framework would be helpful. Currently the manuscript describes activities but does not clearly theorize the engagement approach. (e.g., Hart's Ladder of Participation, Youth Participatory Action Research, CBPR principles, or another engagement framework)

o Response: We appreciate the reviewer’s thoughtful recommendation, and the addition of this information has strengthened our manuscript. Our research process was guided by Youth Participatory Action Research. We have added this sentence to the methods section, “The recruitment and maintenance of our study’s YAB was guided by the three key principles of Youth Participatory Action Research (YPAR): 1) this study is informed by the lived experiences TGD youth, 2) YAB members are collaborators on all aspects of this qualitative study, and 3) the purpose of our qualitative study on TGD youth research participation is to advance research that improves the lives of TGD youth.(16)”

• See lines 104-108 of the clean manuscript.

- A section or thoughts on reflexivity. While information is added on the PI, there is little beyond the facts and not much to discuss how power dynamics were approached, how differences in age, professional status, race, socioeconomic status, and education may have influenced interactions; how the team mitigated tokenism and power imbalances

o Response: We agree with the reviewer that this is very important information. We have added a paragraph within the Engagement subsection to describe the steps we took to address power imbalances between the YAB and other research team members.

• See lines 318-330 of the clean manuscript

- What changed because youth were involved,

o Response: Throughout the manuscript we have noted what is unique to youth advisory board members. For example, for YAB members under 18 years of age, we required legal guardian consent. We gave potential YAB members two separate opportunities to showcase their interest in skills, an interview and a follow-up task because many youth have not participated in formal interviews. We also required flexibility due to many youth members juggling responsibilities of being students. This included additional one-on-one support for one youth member and a delay in coding due to their studying for finals. Another example is applying youth chosen topics (anime and video games) into practice coding sessions.

- How youth themselves evaluated the experience.

o Response: YAB members provided their feedback through one-on-one feedback sessions with the research coordinator and a REDCap survey that included the Research Engagement Survey Tool (REST) and open-ended questions about their participation and our research processes.

• See lines 306-308, 326-328 of the clean manuscript

- Abstract: Partnered with - was the advisory group already in existence and if not how was it formed and recruited? (I see you talk about the recruitment and creation of the advisory group in the methods section, but would be helpful to have a slight nod to it or brief information in abstract since creation of a YAB is different than using a previously created YAB)

o Response: We have edited the sentence to clarify that we developed this YAB as opposed to partnering with an already established YAB. The sentence now reads, “The Research on Identity Specific Eating Disorder Symptoms (RISES) Project developed, partnered with, and maintained a youth advisory board of five transgender, non-binary, and gender-diverse youth in all stages of a qualitative study, including qualitative analysis.”

• See lines 38-41 of the clean manuscript

- Introduction: line 50 - add citation

o Response: We have added a citation.

• See the citation for the second sentence of the first paragraph of the introduction.

- Methods: YAB demographics would be nice

o Response: We have added the YAB’s age, race/ethnicity, gender identity, and state.

• See lines 150-154 of the clean manuscript

- Methods: Were trainings in addition to the 1-2 YAB meetings per month or some months the meetings were trainings?

o Response: These trainings were included in the 1-2 YAB meetings per month. We added a sentence to clarify this.

• See line 159 of the clean manuscript

- Methods: Qualitative analysis process line 206 - was the discussion with the YAB or research team?

o Response: These discrepancy discussions were with the YAB members/mentors. We have added this to qualitative analysis section.

• See lines 217-218 of the clean manuscript

- For all of your lessons learned - how would you recommend someone else utilize these lessons?

o Response: We were intentional on not listing specific recommendations since we acknowledge that high levels of youth engagement in research rely on resources, specifically time and money. Because of this, we provided a variety of examples describing how we maintained high levels of engagement throughout our qualitative analysis process so that readers can consider applying varying levels of youth engagement to their research. We have modified our closing paragraph to reflect this. It now reads, “We acknowledge that youth-engaged research methods are resource intensive and require flexibility, but there are 1) clear benefits to this commitment and 2) varying levels of youth engagement that can be applied to research. We hope that the lessons we have learned in our qualitative study can be adapted to the unique resources and needs of different types of research studies involving youth. We will continue to learn from our YAB and apply the lessons learned to future research protocols with our current and expanding YAB.”

• See lines 374-380 of the clean manuscript

- Communication: line 251 - I wonder if you can speak more to the joint benefit of YAB - while they were informing the research they were also building community amongst themselves with support from allies in positions of power

o Response: Thank you for this special point. We have added a sentence to our closing paragraph about this. Our closing paragraph now reads, “Our partnership with a TGD YAB throughout qualitative analysis strengthened the rigor and validity of our results, and cultivated research skills and professional development of five TGD youth. We were also given the unique opportunity to create a research environment where academicians supported five TGD youth who also provided support for each other during a politically difficult year for TGD communities.”

• See lines 370-374 of the clean manuscript

Attachment Submitted filename: Response to Reviewers.7.10.docx

# Editor report: Decision Letter 1

**DOI:** 10.1371/journal.pone.0355110.r003

**Authors:** Branko Aleksic

17 Jul 2026

Lessons Learned From Youth-Engaged Research Methods to Conduct Qualitative Analysis with a Transgender and Gender-Diverse Youth Advisory Board

PONE-D-26-08402R1

Dear Dr. Pham,

We’re pleased to inform you that your manuscript has been judged scientifically suitable for publication and will be formally accepted for publication once it meets all outstanding technical requirements.

Within one week, you’ll receive an e-mail detailing the required amendments. When these have been addressed, you’ll receive a formal acceptance letter and your manuscript will be scheduled for publication.

An invoice will be generated when your article is formally accepted. Please note, if your institution has a publishing partnership with PLOS and your article meets the relevant criteria, all or part of your publication costs will be covered. Please make sure your user information is up-to-date by logging into Editorial Manager at Editorial Manager® and clicking the ‘Update My Information' link at the top of the page. For questions related to billing, please contact billing support.

If your institution or institutions have a press office, please notify them about your upcoming paper to help maximize its impact. If they’ll be preparing press materials, please inform our press team as soon as possible -- no later than 48 hours after receiving the formal acceptance. Your manuscript will remain under strict press embargo until 2 pm Eastern Time on the date of publication. For more information, please contact onepress@plos.org.

Kind regards,

Branko Aleksic, MD, PhD

Academic Editor

PLOS One

Additional Editor Comments (optional):

The authors have adequately addressed the reviewers' comments and substantially improved the manuscript. The revision strengthens the methodological framework by incorporating a Youth Participatory Action Research (YPAR) framework, provides greater transparency regarding reflexivity, power dynamics, and youth engagement, and clarifies several methodological aspects of the study. The additional details on the Youth Advisory Board, qualitative analysis procedures, and participant engagement enhance the rigor and practical value of the manuscript. I believe the revised version is suitable for publication in its current form.

Reviewers' comments:

# Editor report: Acceptance letter

**DOI:** 10.1371/journal.pone.0355110.r004

**Authors:** Branko Aleksic

PONE-D-26-08402R1

PLOS One

Dear Dr. Pham,

I'm pleased to inform you that your manuscript has been deemed suitable for publication in PLOS One. Congratulations! Your manuscript is now being handed over to our production team.

At this stage, our production department will prepare your paper for publication. This includes ensuring the following:

* All references, tables, and figures are properly cited

* All relevant supporting information is included in the manuscript submission,

* There are no issues that prevent the paper from being properly typeset

You will receive further instructions from the production team, including instructions on how to review your proof when it is ready. Please keep in mind that we are working through a large volume of accepted articles, so please give us a few days to review your paper and let you know the next and final steps.

Lastly, if your institution or institutions have a press office, please let them know about your upcoming paper now to help maximize its impact. If they'll be preparing press materials, please inform our press team within the next 48 hours. Your manuscript will remain under strict press embargo until 2 pm Eastern Time on the date of publication. For more information, please contact onepress@plos.org.

You will receive an invoice from PLOS for your publication fee after your manuscript has reached the completed accept phase. If you receive an email requesting payment before acceptance or for any other service, this may be a phishing scheme. Learn how to identify phishing emails and protect your accounts at https://explore.plos.org/phishing.

If we can help with anything else, please email us at customercare@plos.org.

Thank you for submitting your work to PLOS ONE and supporting open access.

Kind regards,

PLOS ONE Editorial Office Staff

on behalf of

Dr Branko Aleksic

Academic Editor

PLOS One
